# Metagenomic Profiling of Internationally Sourced Sewage Influents and Effluents Yields Insight into Selecting Targets for Antibiotic Resistance Monitoring

**DOI:** 10.1021/acs.est.4c03726

**Published:** 2024-09-04

**Authors:** Emily Garner, Ayella Maile-Moskowitz, Luisa F. Angeles, Carl-Fredrik Flach, Diana S. Aga, Indumathi Nambi, D. G. Joakim Larsson, Helmut Bürgmann, Tong Zhang, Peter J. Vikesland, Amy Pruden

**Affiliations:** † Wadsworth Department of Civil and Environmental Engineering, West Virginia University, Morgantown, West Virginia 26505, United States; ‡ Department of Civil and Environmental Engineering, Virginia Tech, Blacksburg, Virginia 24061, United States; § Department of Chemistry, University at Buffalo, Buffalo, New York 14260, United States; ∥ Institute of Biomedicine, Department of Infectious Diseases, Centre for Antibiotic Resistance Research in Gothenburg (CARe), University of Gothenburg, Västra Götaland, SE-405 30 Gothenburg, Sweden; ⊥ Department of Civil Engineering, Indian Institute of Technology, Madras, Chennai 600036, India; # Eawag: Swiss Federal Institute of Aquatic Science and Technology, Kastanienbaum CH-6047, Switzerland; ¶ Department of Civil Engineering, The University of Hong Kong, Pokfulam 999077, Hong Kong

**Keywords:** antibiotic resistance, resistome, wastewater treatment, metagenomics, monitoring

## Abstract

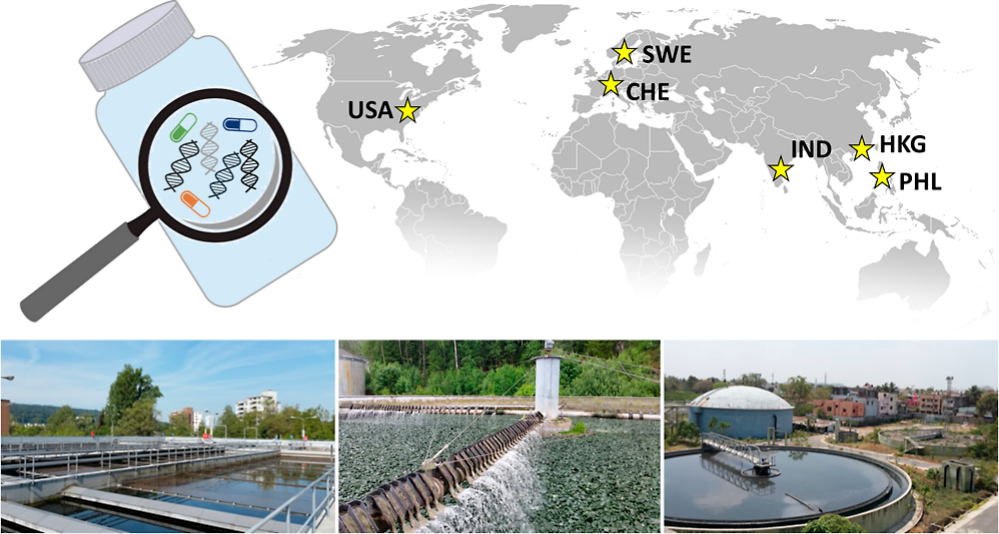

It has been debated whether wastewater treatment plants (WWTPs) primarily act to attenuate or amplify antibiotic resistance genes (ARGs). However, ARGs are highly diverse with respect to their resistance mechanisms, mobilities, and taxonomic hosts and therefore their behavior in WWTPs should not be expected to be universally conserved. We applied metagenomic sequencing to wastewater influent and effluent samples from 12 international WWTPs to classify the behavior of specific ARGs entering and exiting WWTPs. In total, 1079 different ARGs originating from a variety of bacteria were detected. This included ARGs that could be mapped to assembled scaffolds corresponding to nine human pathogens. While the relative abundance (per 16S rRNA gene) of ARGs decreased during treatment at 11 of the 12 WWTPs sampled and absolute abundance (per mL) decreased at all 12 WWTPs, increases in relative abundance were observed for 40% of the ARGs detected at the 12th WWTP. Also, the relative abundance of mobile genetic elements (MGE) increased during treatment, but the fraction of ARGs known to be transmissible between species decreased, thus demonstrating that increased MGE prevalence may not be generally indicative of an increase in ARGs. A distinct conserved resistome was documented in both influent and effluent across samples, suggesting that well-functioning WWTPs generally attenuate influent antibiotic resistance loads. This work helps inform strategies for wastewater surveillance of antibiotic resistance, highlighting the utility of tracking ARGs as indicators of treatment performance and relative risk reduction.

## Abstract 2

Metagenomic sequencing across a transect of wastewater treatment plant influents and effluents reveals which antibiotic resistance genes (ARGs) tend to decrease or amplify during treatment, informing surveillance and risk reduction strategies.

## Introduction

Bacterial resistance to antibiotics is a serious global health threat and the role of water, sanitation, and hygiene in controlling its spread is increasingly recognized.^1^ Expansion and improvement of sewage collection and treatment via a wastewater treatment plant (WWTP) is one means of curbing the spread of resistant microorganisms via environmental pathways.^2^ WWTPs often employ biological treatment, such as activated sludge, wherein aeration is applied to stimulate organic matter biodegradation by flocs of heterotrophic bacteria, which are subsequently removed via settling with the aqueous effluent returned to the environment. WWTPs are highly effective in reducing the concentrations (cells/L) of bacteria leaving the plant, including pathogens. Removal of antibiotic resistant bacteria (ARB), such as *Escherichia coli* and *Enterococcus* spp., by activated sludge treatment have likewise been reported in the range of 2–5-log,^3,4^ but ARB of clinical concern can persist in WWTP effluents and pose health risks.^5^ There remains a lack of consensus about whether WWTPs act to increase versus attenuate the relative abundance, i.e., abundance normalized to a marker of the total bacterial population such as the 16S rRNA gene, of ARB or antibiotic resistance genes (ARGs).^5−7^ Shifts in relative abundances of ARB and ARGs could reflect selective pressures occurring within the WWTP, but also will be strongly shaped by microbial ecological shifts that reflect aeration of the secondary bioreactor.

Previous research has demonstrated that the relative abundance of some ARGs can be reduced during wastewater treatment, such as *erm*B,^8^*bla*_OXA-58_,^9^*tet*C,^9^ and *tet*M.^9^ Heterotrophic bacteria resistant to vancomycin, gentamicin, erythromycin, cephalexin, tetracycline, and sulfadiazine have been reported to decline.^10^ Yet, other studies have found minimal impact or even enrichment of ARGs, such as *van*A,^8^*bla*_VIM_,^8^ and *bla*_SHV-34_.^9^ Ju et al. observed increased relative abundance of 12 resistance classes, but also biocide and heavy metal resistance classes.^11^ Another study reported increases in drug-resistant *Acinetobacter* spp.^12^ Several studies have documented differences in removal or enrichment of ARB and ARGs among WWTPs.^9,11^ Further, critical differences in social, clinical, regulatory, and environmental factors around the world are expected to influence the types and magnitudes of ARB and ARGs in influent sewage and thus the efficacy of the WWTP as a barrier for ARB/ARG removal.^13−15^

There are several drivers that may enrich ARB and ARGs within a WWTP. For example, horizontal gene transfer (HGT) facilitates transfer of ARGs to new cell hosts that grow at higher rates in the activated sludge environment. HGT is particularly relevant in the parts of the WWTP with high bacterial cell density (e.g., activated sludge)^16^ and might be exacerbated by sewage coconstituents; such as organic compounds, residual antibiotics, and heavy metals, which have been shown to increase rates of conjugation or natural transformation.^17−20^ Biological wastewater processes have been characterized as housing a high co-occurrence of bacterial species capable of donating their ARGs via HGT, along with corresponding mobilizing agents, such as insertion sequences.^21^ Selection, HGT, or induction of mutations that increase resistant microbiota have been associated with constituents commonly present in wastewater; such as antibiotics, metals, herbicides, and antidepressants.^22−25^ Shifts in microbial community structure have been reported to be a key driver of shifts in ARGs in soil,^26^ and this may also be an important driving force in WWTPs.^11^

High throughput metagenomic DNA sequencing can be used to efficiently profile and compare known ARGs across WWTPs.^13,14,27^ However, with over 3500 different ARGs documented to date,^28^ efforts are needed to establish a baseline understanding of which ARGs are present in the influent versus effluent, which tend to attenuate versus increase during treatment, and whether influent ARG composition significantly affects treatment outcomes. Such knowledge will help improve our understanding of the extent to which WWTPs effectively act as a barrier to the spread of antibiotic resistance as well as to identify anomalies that could indicate problems with treatment or flag emerging public health concerns.

The objective of this study was to broadly categorize ARGs according to their reduction or enrichment during wastewater treatment. The following key hypotheses were tested: (1) relative (per 16S rRNA gene) and absolute (per mL) abundance of total ARGs decrease as a result of wastewater treatment; (2) the final effluent resistome composition mirrors regional patterns previously documented in the influent;^14^ (3) removal efficiencies for individual ARGs vary widely across and within resistance classes and WWTPs; (4) wastewater treatment reduces the abundance of ARGs carried by bacteria causing human disease; (5) biological wastewater treatment increases ARG diversity in the final effluent relative to the influent; (6) wastewater treatment leads to a reproducible shift in resistome composition across geographically diverse WWTPs. By leveraging metagenomic sequencing across 12 diverse WWTPs from six countries in three continents generated using a consistent field collection and analysis protocol, this study provides insight into the extent to which treatment impacts on the resistome are conserved globally across facilities and proposes specific metrics for comparing treatment efficacy and identifying potential human health hazards. While previous studies have used metagenomic sequencing to comprehensively profile the antibiotic resistome in sewage^13,29^ or to monitor changes across individual treatment trains,^4,27^ this study is the first to examine the effects of biological wastewater treatment on the resistome across a broad range of international facilities.

## Materials and Methods

### Site Description and Sample Collection

Sampling was conducted at two WWTPs from each of six countries (India (IND), Hong Kong, China (HKG), Philippines (PHL), United States of America (USA), Switzerland (CHE), and Sweden (SWE)), between March 2016 and January 2017. All WWTPs employed secondary treatment, with capacities ranging from 2.6 to 66 million gallons per day. Both Hong Kong plants (HKG-1, HKG-2) and one USA plant (USA-1) employed UV-disinfection of effluent; one Philippines WWTP (PHL-1), one USA WWTP (USA-2), and both India plants (IND-1 and IND-2) disinfected using free chlorine; and one Swiss WWTP (CHE-2) disinfected via ozonation followed by sand filtration. The remaining four WWTPs did not disinfect final effluent. Additional characteristics of the sampled WWTPs have been published previously,^14^ along with a detailed examination of their influent resistomes, and are described in additional detail in Table S1. Sample collection and processing were conducted using standardized protocols validated for sample preservation and stability during international shipment.^30,31^ Influent and final effluent grab samples for molecular analysis were collected at each WWTP in sterile polypropylene containers and samples for antibiotic analysis were collected in acid-washed, baked amber glass bottles.

### DNA Extraction and Quantitative Polymerase Chain Reaction

Samples were collected, processed, preserved and shipped according to Li et al.^31^ Within 12 h of collection, triplicate aliquots of each sample were concentrated onto 0.22 μm mixed cellulose ester membranes (Millipore, Billerica, MA) until clogging. In cases where clogging occurred prior to the maximum amount of water collected passing through the filter, less than the full volume was filtered. The actual volume filtered was recorded and used for normalization. Filters were preserved in 50% ethanol and shipped to Virginia Tech on ice packs. Upon arrival, filters were frozen at −20 °C until DNA extraction. Filters were aseptically torn into 1 cm^2^ pieces using sterile forceps and transferred to extraction tubes. DNA was extracted using the FastDNA SPIN Kit for Soil (MP Biomedicals, Solon, Ohio). Quantitative polymerase chain reaction (qPCR) was used to quantify 16S rRNA genes and the *sul*1 sulfonamide ARG in triplicate reactions using previously published primers (Table S2).^32,33^ Triplicate standard curves of 10-fold serial diluted standards of each target gene ranging from 10^1^ to 10^7^ gene copies/μL were included on each 96-well plate, along with a triplicate negative control.

### Metagenomic Sequencing and Analysis

Composite samples were prepared by pooling triplicates by equal DNA mass. Composites were prepared for sequencing using TrueSeq library preparation (Illumina, San Diego, CA) and sequenced on an Illumina HiSeq 2500 using 2 × 100 paired-end reads at the Virginia Tech Biocomplexity Institute Genomic Sequencing Center (Blacksburg, VA). Reads were uploaded to MetaStorm,^34^ where quality filtering was performed using Trimmomatic according to default parameters.^35^ Reads were assembled in MetaStorm using IDBA-UD.^36^ Reads and scaffolds were annotated using default parameters in MetaStorm against the Comprehensive Antibiotic Resistance Database (CARD; version 2.0.1) to identify ARGs,^37^ the Silva rRNA database for 16S rRNA genes,^38^ and the ACLAME database (version 0.4) for mobile genetic elements (MGEs).^39^ Taxonomy was assigned to reads using MetaPhlan2.^40^ To calculate relative ARG abundance, ARG read counts were normalized to 16S rRNA gene counts.^41^ To calculate absolute abundance, 16S rRNA gene normalized abundances were multiplied by 16S rRNA gene abundances per mL, as measured by qPCR. Mobile ARGs were defined based on inclusion in the ResFinder database (version 2.1),^42^ and were manually curated from CARD annotations. Assembled scaffolds were uploaded to NanoARG^43^ for identification of ARGs, MGEs, and metal resistance genes using default parameters. Metagenomes are publicly available via National Center for Biotechnology Information’s BioProject PRJNA527877.

### Antibiotic Analysis

Sample analysis for antibiotics was conducted as previously described and antibiotic concentrations in these samples have been previously published.^30^ Briefly, wastewater samples (0.5 L) were acidified and filtered using 0.45 μm glass microfiber filters to remove microorganisms and particulate matter. Na_2_EDTA (2 mL, 5% v/v in water) and surrogate standards (50 μL of 1000 μg/L surrogate mix solution) were added to each sample. Solid phase extraction was performed by conditioning Oasis HLB cartridges with acetonitrile and deionized water before the water samples were loaded at a rate of 3–5 mL/min. Cartridges were dried under vacuum and shipped to the University at Buffalo for elution and liquid chromatography with tandem mass spectrometry (LC–MS/MS) analysis using an Agilent 1200 LC system (Palo Alto, CA).

### Statistical Analysis

Differences in ARG abundance between groups were tested using a Wilcoxon rank sum test in R.^44^ Percent mobility was defined as described by Ju et al., determined by calculating the percentage of scaffolds containing both ARGs and MGEs out of the total number of ARG-containing scaffolds.^11^ Correlations between antibiotics and ARGs were tested using a Spearman rank sum test in R.^44^ To compare resistome profiles across samples, ARG abundances were standardized to a percentage of the total ARGs from each sample and a Bray–Curtis resemblance matrix was generated in Primer-E (version 6.1.13). Nonmetric multidimensional scaling (NMDS), analysis of similarities (ANOSIM), and similarity clustering were conducted in Primer-E. Shannon diversity of microbial genera and ARGs, as well as symmetric Procrustes and PROTEST analysis were conducted in R using vegan.^45^ Linear discriminant analysis Effect Size (LEfSe) was applied to identify ARGs that best explain differences between sample groups.^46^ Figures were produced using ggplot2^47^ and Microsoft Excel.

## Results

### Detection of ARGs and Quantitative Assessment of Metagenomic Data

**Figure 1:**
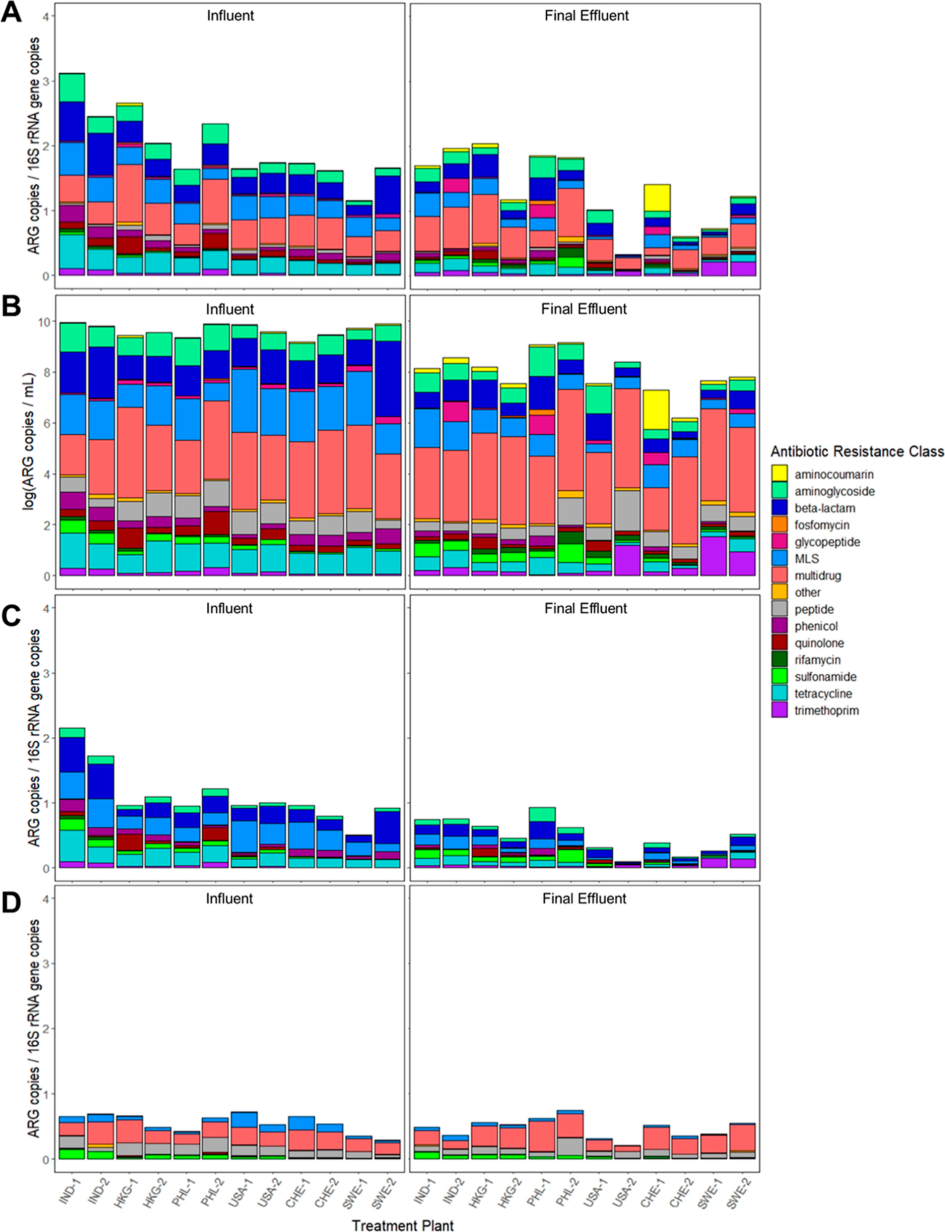
ARGs in WWTP influent and effluent based on (A) relative abundance annotated against the CARD database via MetaStorm and normalized to 16S rRNA genes, (B) absolute abundance of ARGs annotated against the CARD database via MetaStorm and normalized to sample volume, (C) potentially mobile ARGs, identified as those included in the ResFinder database of acquired ARGs, and (D) ubiquitous ARGs (i.e., detected in all samples).

Metagenomic sequencing yielded over 326 million paired-end reads across all samples, with an average of 1.36 × 10^6^ reads per sample (range: 0.92–1.78 × 10^6^). Across the data set, 1079 different ARGs were identified, with up to 554 different ARGs detected in a single sample (SWE-2 influent; Table S3). The most abundant ARG classes in the influent, on average, were multidrug, macrolide-lincosamide-streptogramin (MLS), beta-lactam, tetracycline, and aminoglycoside (Figure 1A). Similarly, many of the same classes of ARGs dominated the effluent resistome, of which multidrug, MLS, beta-lactam, aminoglycoside, and peptide were most abundant. Relative abundance of *sul*1 normalized to 16S rRNA genes correlated with absolute abundances of *sul*1 genes per unit volume measured using qPCR (R^2^ = 0.48, *p* = 0.031) (Figure S1). The strong alignment of these results supports the use of subsequent quantitative comparisons made via metagenomic data. However, results are presented in terms of both relative abundance (per 16S rRNA gene copies) and absolute abundance (per mL; Figure 1) to provide insight into the effect of changing overall microbial concentrations on the observed resistome.

### Impact of Treatment on Total ARG Abundance

While many ARG classes remained dominant from the influent to the final effluent, the overall relative abundance of the summed total ARGs (total ARGs) decreased at 11 of 12 WWTPs (*p* = 0.01 across the data set). Only one WWTP, PHL-1, exhibited an increase in relative total ARG during treatment. Absolute abundance (per mL) of total ARGs exhibited a similar trend to that observed for relative abundance, decreasing at all 12 WWTPs by ∼0.5–3.5 log (Figure 1B). Hereafter, we focus on relative abundance as an indicator of the tendency of ARGs to be enriched or attenuated across WWTP microbial communities.

### Patterns in the Fate of Individual ARGs

**Figure 2:**
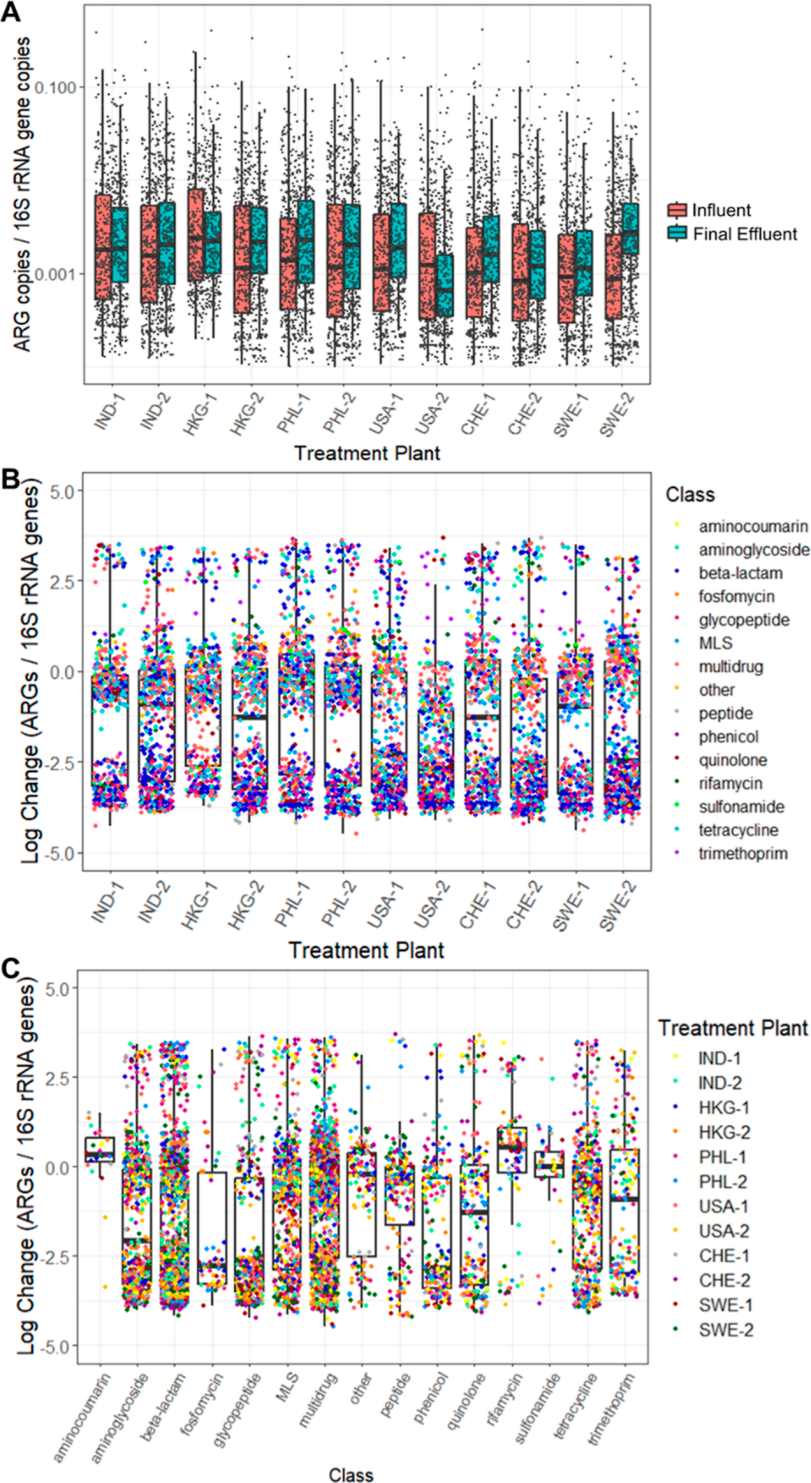
(A) Relative abundance of all detected ARGs in each WWTP’s influent vs final effluent. (B) Log change in relative abundance of individual ARGs during treatment at each WWTP. (C) Log change in relative abundance of individual ARGs during treatment by antibiotic resistance class.

The relative abundance of each ARG is provided in Table S3. While both the relative and absolute abundance of total ARGs decreased consistently during treatment, many individual ARGs increased in relative abundance from the influent to final effluent (Figure 2A). Notably, the greatest percentage of ARGs (40.0%) increased at the PHL-1 WWTP, which was also the only WWTP that did not yield a decrease in relative total ARG abundance. Increases in ARG abundance at the remaining WWTPs ranged from 8.2 to 32.8%: 32.8 and 22.7% of detected ARGs increased in Swiss WWTPs, 32.5 and 26.3% of ARGs in Hong Kong WWTPs, 21.1 and 25.6% in Indian WWTPs, 28.2% in the remaining Philippines WWTP, 24.3 and 31.1% in Swedish WWTPs, and 24.4 and 8.2% in USA WWTPs. Increases in abundance of these enriched ARGs were striking in many instances, sometimes reaching >3-log enrichment (Figure 2B). Across all ARGs and all WWTPs, there was a notable bimodal distribution in the log change in relative abundance, as evidenced by clustering of datapoints in Figure 2B,C: 45.4% of data points indicated between −4.0 and −3.0 log change in relative abundance of ARGs, while 40.3% were between −1.0 and +1.0 log. This bimodal distribution was driven by two common scenarios: (A) ARGs abundant in influent wastewater, but undetectable in the final effluent, and (B) ARGs that were undetectable in either the influent or final effluent and only detected at low levels in the other.

**Figure 3:**
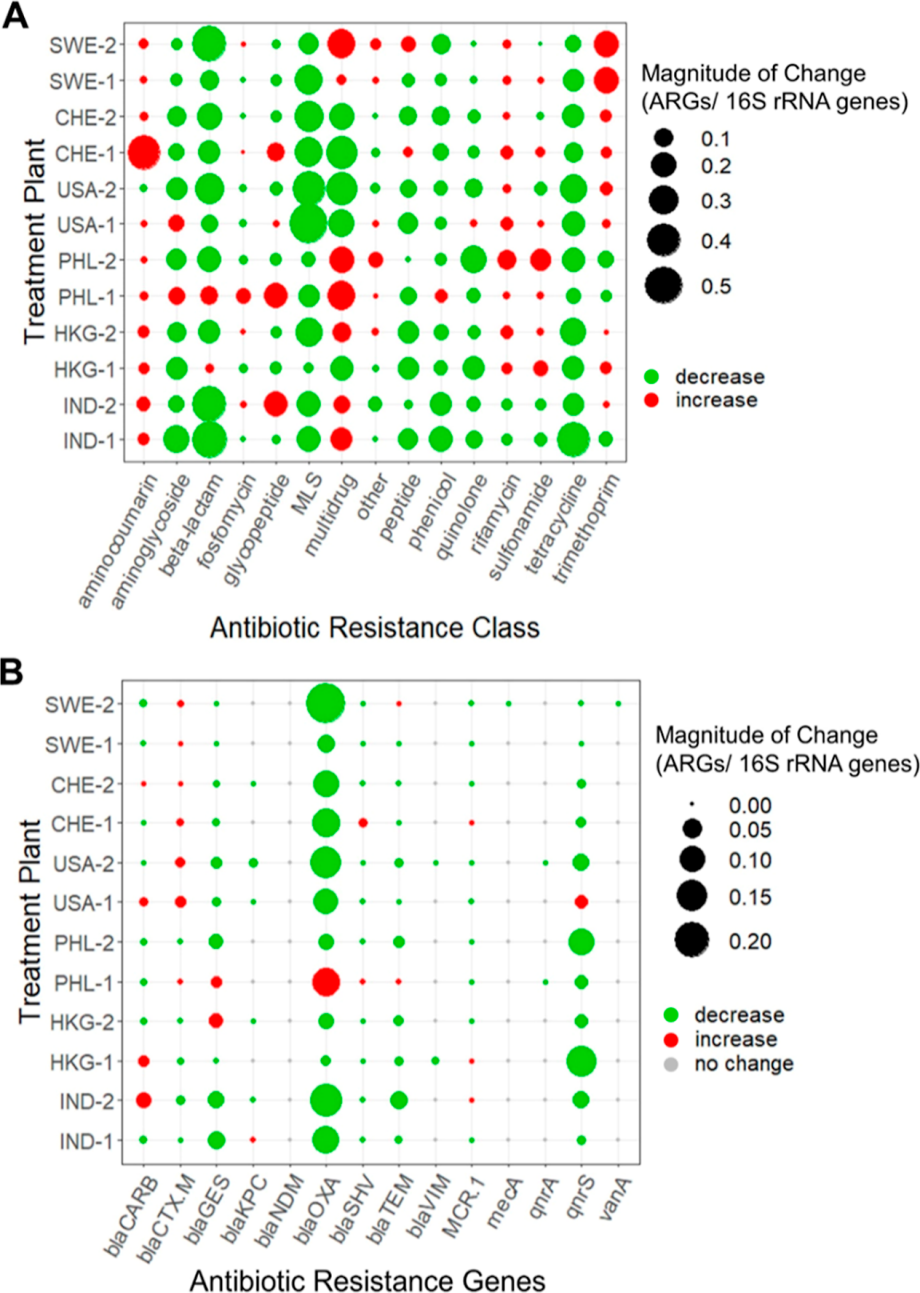
Change in abundance following treatment of (A) ARGs according to class of resistance and (B) select clinically relevant ARGs.

Some notable trends in reduction or enrichment of ARGs were observed as a function of resistance class (i.e., the class of antibiotics to which ARGs encode resistance; Figure 3A). For example, the relative abundance of total tetracycline and MLS ARGs decreased with treatment across all WWTPs. Aminoglycoside, beta-lactam, peptide, phenicol, and quinolone ARGs were reduced in relative abundance at nearly all WWTPs. On the contrary, aminocoumarin, multidrug, rifamycin, sulfonamide, and trimethoprim ARGs increased in relative abundance at the majority of WWTPs. While these general trends are notable, it is important to point out that substantial variations in changes were observed among individual ARGs within each class (Figure 2C).

Across all WWTPs, there was a significant increase in ARG diversity from the influent to the final effluent (Wilcox, *p* = 0.0005; Figure S2A), consistent with the biological reactors harboring a distinct microbial community that carry a distinct array of ARGs that are additionally detected in final effluents. It was noted that some ARGs that were highly abundant in the influent were among those that experienced the greatest overall reduction during treatment. Twenty-two ARGs fell within both the 15th percentile of the highest influent concentrations and the 15th percentile of greatest reductions in relative abundance during treatment (Figure S3): two aminoglycoside (APH(3″)-lb, APH(6)-ld), two beta-lactam (*Cfx*A4, *Cfx*A6), six MLS (*Erm*B, *Erm*F, *mef*A, *mel*, *mph*D, *msr*E), four multidrug (*ade*J, *ade*K, *ade*M, CRP), one peptide (*pmr*E), one phenicol (*cat*), two quinolone (*qac*H, *Qnr*S2), one sulfonamide (*sul*1), and three tetracycline (*tet*(39), *tet*Q, *tet*W) ARGs. While decreases in *sul*1 concentrations were notable, decreasing by 0.00981 ± 0.02380 gene copies per 16S rRNA gene across all plants, the noted increase in sulfonamide ARGs at the class level (Figure 1A,B) was largely driven by *sul*2 and *sul*4, with increases of 0.01435 ± 0.03717 and 0.00461 ± 0.00810 gene copies per 16S rRNA gene, respectively.

### Conserved and Differential Aspects of Influent and Effluent Resistomes

The relative similarities in ARG profiles across globally distributed WWTPs is noteworthy (Figures 1A,B). The core resistome was evaluated to identify the portion of the resistome that is “ubiquitous” across all influent and effluent sites (Figure 1D). The core influent resistome included 233 subtype ARGs shared across all influent samples, while the core effluent included 51 subtype ARGs. Overall, 49 ARGs at the subtype level were ubiquitous across all influent and effluent samples. This group was overwhelmingly dominated by multidrug (*n* = 31) ARGs. There were no ARGs at the subtype level that were detected exclusively in the influent or final effluent.

LEfSe analysis was applied to determine which ARGs were the key drivers of differences in the influent and effluent resistomes (Figure S4). Two tetracycline (*tet*Q, *tet*A(46)), three beta-lactam (*Cfx*A6, *Cfx*A4, CTX-M-107), two MLS (*mph*B, *Erm*G), one quinolone (*pat*B), one fosfomycin (*Fos*A5), four multidrug (*lsa*B, *lsa*C, *mdt*O, *ram*A), and one glycopeptide (*van*SG) ARG were key features of the influent resistomes. Key features of the final effluent resistome included two multidrug (*mex*N, *opr*A), one rifamycin (*rph*A), and one trimethoprim ARG (*dfr*A2d).

**Figure 4:**
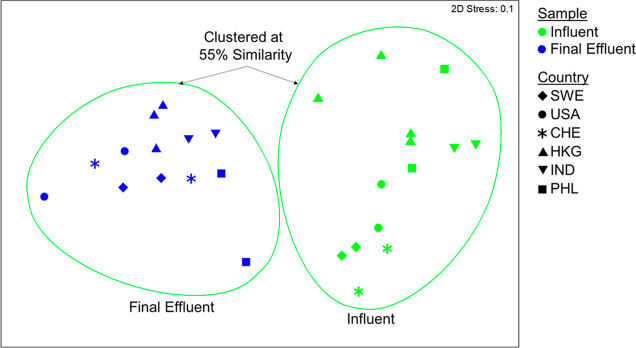
NMDS plot depicting the resistome of influent versus final effluent samples from twelve global WWTPs. Similarity was calculated according to the Bray–Curtis metric. Samples clustered at 55% similarity.

NMDS was used to visualize differences in the resistome composition of each sample (Figure 4). Influent and effluent samples formed distinct clusters, sharing 55% similarity (ANOSIM, *R* = 0.926, *p* < 0.001). Effluent resistomes formed a tighter cluster than influent resistomes, with the PHL-1 effluent an apparent outlier. At a high level, the shared ecology of the biological treatment environment shifted the resistome composition from influent to effluent in a similar manner, regardless of geographical location and initial influent resistome composition.

### Fate of Clinically-Relevant ARGs in the WWTPs

Key clinically relevant ARGs were selected to assess their removal by the 12 WWTPs (Figure 3B). While OXA-type carbapenemases were the most common clinically relevant ARG detected, they were effectively removed at all WWTPs sampled, except the PHL-1 WWTP. GES, SHV, and TEM-type beta-lactam ARGs were also typically removed during treatment, but removal patterns of CARB and CTX-M beta-lactam ARGs varied among WWTPs. New Delhi metallo (NDM) beta-lactamase ARGs were not detected in any influent or effluent sample. MCR-1, which confers resistance to colistin, was detected in both influent and effluent, though at very low abundance (≤0.00144 copies/16S rRNA gene). *qnr*S, a plasmid-mediated quinolone ARG, was effectively removed at 11 of 12 WWTPs, with USA-1 the exception.

**Figure 5:**
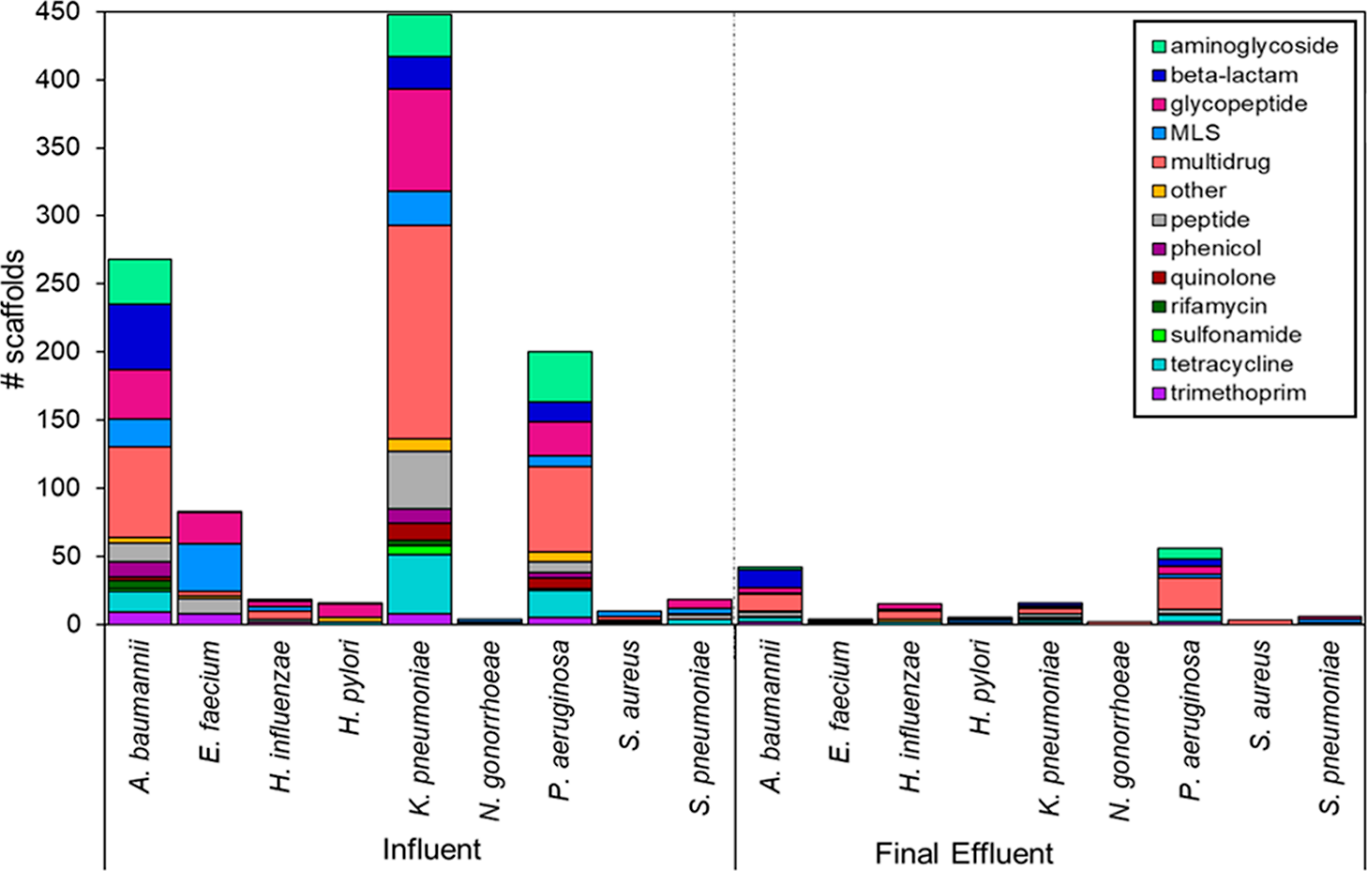
Abundance of ARGs, by class, identified on assembled scaffolds aligning with pathogen reference genomes using default parameters in NanoARG.^43^

A total of 5,740,060 scaffolds were generated, averaging 735 bp in length and 179,377 scaffolds per sample (range: 64,576–256,387). Of these, 1214 scaffolds were associated with the WHO list of priority antibiotic resistant pathogens or the ESKAPE pathogens (i.e., *Enterococcus faecium*, *Staphylococcus aureus*, *Klebsiella pneumoniae*, *Acinetobacter baumannii*, *Pseudomonas aeruginosa*, and *Enterobacter* species), which were further examined for carriage of ARGs using NanoARG (Figure 5).

Multidrug ARGs were found on a substantial percentage of the scaffolds associated with pathogens in the influent and effluent (respectively) for *A. baumannii* (25, 29%), *Haemophilus influenza* (33, 40%), *K. pneumoniae* (35, 25%), *Neisseria gonorrheae* (25, 100%), *P. aeruginosa* (32, 41%), and *S. aureus* (30, 100%). Among the effluent samples, only two scaffolds were annotated as *N. gonorrheae* and three as *S. aureus*, so the 100% carriage of multidrug resistance is likely an artifact of low detection rate. Among the influent scaffolds, 28% annotated as *E. faecium* contained glycopeptide ARGs, a pathogen-resistance combination of high clinical concern. In the effluent, zero of the four scaffolds annotated as *E. faecium* contained glycopeptide ARGs. In contrast, beta-lactam resistance increased from influent to effluent on *A. baumannii* (from 18 to 31%) and *P. aeruginosa* (from 7 to 9%) scaffolds.

All scaffold annotations are provided in Table S4. Scaffolds were further analyzed if they originated from a putative pathogen and contained three or more ARGs, MGEs, metal resistance genes, and, in some cases, repeated detection of conserved regions across multiple samples (Figure 5). Four such assembled regions were derived from *A. baumannii*, two of which conferred resistance to multiple antibiotics and were found in the final effluent of SWE-1 (Figure S5A,D). One scaffold also included multiple transposase genes and genes conferring resistance to silver and zinc. The remaining *A. baumannii* scaffolds (Figure S5B,C) were found in influent samples collected from SWE-1 and PHL-1 and carried four different ARGs each. One assembled region derived from *E. faecium* was particularly noteworthy as the same region was detected in influent samples from CHE-2, SWE-1, SWE-2, and USA-1 (Figure S5E). The region confers resistance to bacitracin, a peptide antibiotic, as well as glycopeptide antibiotics and zinc. This region also contained a conjugative transposon gene. The recurrent detection of this genetic region in different countries suggests that it may be a strain or genetic region globally carried by human populations. The same genetic region was not detected in any final effluent sample. A genetic region associated with *H. influenzae* was derived from the influent of USA-1 (Figure S5F) and carried a transposase as well as ARGs conferring resistance to beta-lactam and aminoglycoside antibiotics. Finally, a genetic region found on a scaffold originating from *P. aeruginosa* in a HKG-1 influent sample (Figure S5G) carried ARGs associated with MLS, peptide, aminoglycoside, and glycopeptide resistance, as well as arsenic resistance, recombinase, and transposase genes.

### Evaluation of Potential Factors Driving Resistome Shifts from Influent to Effluent

#### Potential for HGT

The portion of potentially mobile ARGs ranged from 16.8 to 57.1% of the overall resistome of each sample (Figure 1C). This mobile resistome was dominated by beta-lactam (23.2% of samples on average), MLS (23.1%), and tetracycline (14.6%) ARGs and significantly decreased in relative abundance as a result of treatment (*p* ≤ 0.0001), from 1.10 to 0.49 ARGs per 16S rRNA gene, on average. In influent samples, the most abundant mobile ARGs were *msr*E (MLS), *tet*Q (tetracycline), *mph*D (MLS), *cat* (phenicol), *sul*1 (sulfonamide), *Erm*F (MLS), *Cfx*A4 (MLS), *Cfx*A6 (MLS), and *qnr*S (quinolone), in descending order. In final effluent, the most abundant mobile ARGs were *sul*1 (sulfonamide), *dfr*B6 (trimethoprim), *sul*2 (sulfonamide), *aad*A11 (aminoglycoside), *cat* (phenicol), *msr*E (MLS), *tet*(C) (tetracycline), and *Erm*F (MLS). MGEs were significantly enriched in final effluent samples compared to the influent (Wilcox, *p* = 0.002316) (Figure S6). This is in contrast to the above observation that the mobile fraction of the resistome was reduced during treatment.

The frequencies of detection of all ARGs on MGE-associated scaffolds are presented in Table S5. The ARGs most frequently found on MGE-associated scaffolds were *mac*B (MLS, 13,014 scaffolds), *Tae*A (pleuromutilin, 5565 scaffolds), *evg*S (multidrug, 5372 scaffolds), and *tet*A(48) (tetracycline, 4887 scaffolds). Clinically relevant ARGs found on MGE-associated scaffolds included MCR-1 (peptide, 73 scaffolds), *qnr*S (quinolone, 18 scaffolds), and a variety of OXA ARGs (beta-lactam, 479 scaffolds). *sul*1, a sulfonamide ARG commonly targeted as an indicator of mobile resistance, was found on 62 MGE-associated scaffolds. Percent mobility (i.e., percentage of scaffolds containing both ARGs and MGEs out of the total number of ARG-containing scaffolds) among samples ranged from 9.9 to 13.6%, with no significant difference between influent and final effluent or between geographic regions (Table S6). Excluding ARGs with only a single scaffold occurrence, the ARGs with the highest percent mobility included *ole*B (MLS, 76.1%), *srm*B (MLS, 74.0%), *car*A (MLS, 69.1%), *Cfx*A2 (beta lactam, 68.8%), *vga*E (multidrug, 67.8%), *sul*1 (sulfonamide, 66.7%), and *msr*C (MLS, 66.7%). Other ARGs of note with high percent mobility include *tet*W (tetracycline, 39.2%), *sul*2 (sulfonamide, 34.3%), TEM (beta-lactam, 16.7%), *Qnr*S6 (quinolone, 14.3%), OXA (beta lactam, 12.3%), MCR-1 (peptide, 11.8%), and VIM (beta lactam, 11.1%) (Table S7).

#### Potential for Selection by Antibiotics During Treatment

Concentrations of sulfonamide, macrolide, quinolone, and tetracycline antibiotics were determined from influent and effluent samples collected at each WWTP (Figures S7 and S8).^30^ Total antibiotic concentrations in influent varied widely from 171 (SWE-2) to 47,978 ng/L (IND-1). Macrolide antibiotics were particularly prevalent in India and Hong Kong WWTPs, tetracyclines were most abundant in Hong Kong WWTPs, and the USA and Swiss WWTPs were dominated by quinolones and sulfonamides. Treatment reduced total antibiotics at 11 of 12 WWTPs, with total removal ranging from 30 to 98%. Total antibiotic concentrations increased at one WWTP, PHL-2, from 1918 to 2023 ng/L, though these concentrations were much lower than those measured in the influent at other Asian WWTPs.

To examine the potential for coselection, co-occurrence of ARGs on scaffolds was examined. The complete list of ARGs co-occurring on assembled scaffolds is presented in Table S8. *van*R and *van*S were the most common ARGs to occur on the same scaffold (194 co-occurrences), which is consistent with these ARGs being components of a vancomycin-resistance operon. A variety of co-occurrences of ARGs that are not part of the same operon were observed, which can potentially be coselected by selective agents. Many of the most abundant of these co-occurred with *van*R and *van*S. *cpx*R (multidrug), *Pvr*R (aminoglycoside), and *mtr*A (multidrug) were associated with *van*S (42, 34, 26 co-occurrences respectively) and *bae*S (multidrug), *sme*S (multidrug), *Pvr*R (aminoglycoside), and *cpx*A (multidrug) were associated with *van*R (31, 30, 28, 25 co-occurrences respectively). This suggested that resistance to glycopeptides may potentially be coselected for by a variety of other antibiotic compounds.

#### Influence of Microbial Community on Resistome

The microbial community experienced marked shifts as a result of treatment that were largely conserved across the sampled WWTPs (Figure S9A). Influent samples were dominated by Gammaproteobacteria, Clostridia, and Bacteriodia, while final effluent samples were primarily dominated by Betaproteobacteria and Gammaproteobacteria. There was a moderate concordance between the structure of the microbial community and that of the resistome (PROTEST: Procrustes Sum of Squares (*m*_12_^2^) = 0.7326, correlation in symmetric Procrustes rotation = 0.5171, significance = 0.004.; Figure S10). In addition, numerous specific taxonomic classes were significantly and positively correlated with several ARG classes (Figure S9B). While ARG diversity consistently increased from the influent to the effluent of each WWTP, this trend did not appear to be driven by a change in microbial diversity, as there was no consistent increase in microbial diversity (Figure S2B).

## Discussion

Metagenomic sequencing provided insight into the occurrence and fate of a wide array of ARGs across 12 globally distributed WWTPs in six countries. Detailed analysis aided in delineating typical responses of specific ARGs and ARG classes to biological treatment. Consistent with our hypothesis (1), total ARG relative abundance decreased in 11 of 12 WWTPs sampled, and corresponding absolute abundances decreased at all 12 WWTPs. However, in alignment with our hypothesis (3), the removal efficiencies for individual ARGs varied widely across and within resistance classes and WWTPs. Consistent with our hypothesis (4), clinically relevant ARGs generally decreased during treatment at the majority of WWTPs, consistent with the findings of previous studies.^4,27^ Others have recently reported striking trends in resistome compositions of raw sewage collected globally,^13,14^ noting abundance of total and clinically relevant ARGs are higher in Asian than US/European sewages. The present study expands this finding by noting the same pattern is retained in the corresponding WWTP final effluents, even while globally sourced effluent resistomes as a group were distinct from influents, which confirms our hypothesis (2). While this study did not address temporal variability in the resistome composition at each site, several previous studies have demonstrated that sewage resistomes are relatively stable over time, with minimal variation in the broader resistome composition during the short-term.^27,29^

WWTP PHL-1 was consistently noted as an outlier herein. For example, relative abundance of total ARGs as well as several classes of resistance that decreased across the majority of WWTPs increased during treatment at PHL-1, including aminoglycoside, beta-lactam, fosfomycin, glycopeptide, and phenicol resistance. Similarly, beta-lactam ARGs of GES, OXA, SHV, and TEM types all increased during treatment at PHL-1, despite decreases noted at the majority of studied plants. This finding highlights the value in examining changes in relative abundance at multiple levels, including the overall resistome, among antibiotic resistance classes, and at the individual gene level, as PHL-1 was not noted as an outlier when examining the overall resistome (Figure 1). One possible explanation for the atypical effect of PHL-1 on ARGs was that this facility employed an attached growth biofilm reactor for secondary treatment. All other facilities relied primarily on suspended growth processes, with the only other attached growth process examined being a trickling filter treating approximately 30% of the total flow at SWE-1. The ecological processes governing the microbial community composition and ARG profile in biofilm-based systems are unique from activated sludge processes due to factors such as sorption of antibiotics and micropollutants onto the biofilm matrix^48^ and stratification of ARB and ARGs in biofilms with filter depth.^49^

While there was wide variability in the relative and absolute abundance of individual ARGs across the 12 WWTPs, there were also notable similarities. Consistent with our hypothesis (6), the overall resistome shifted in a reproducible manner from the influent to effluent that was conserved across all global sites. Of the 1079 ARGs detected overall, 49 were detected consistently across all samples. These 49 ARGs have limited clinical relevance, as none of them are categorized as Rank 1 ‘current threats’ or Rank 2 'future threats'.^50^ There are likely two drivers for the ubiquity of ARGs that persist in the effluent: (1) they are consistently found in the human gut and are poorly removed via typical secondary wastewater treatment processes or (2) they are widespread in water and other natural environments. Additional study is needed to comprehensively compare WWTP influent and effluent to other relevant environments, including those local to each WWTP, to differentiate which of the ubiquitous ARGs fall into each group. ARGs in the first group could serve as conserved indicators for monitoring the effectiveness of wastewater treatment processes or for assessing antibiotic resistance pollution associated with WWTP effluent. ARGs in the second group would be poorly suited for such a purpose due to their ubiquity even in environments not impacted by WWTPs or human fecal pollution.

Multidrug efflux ARGs dominated the total ARG profiles (Figure 1A) and effluent trends more strongly reflected influent trends when the analysis focused on the Resfinder database of mobile ARGs, which largely excluded this class (Figure 1C). Multidrug efflux pump ARGs are highly conserved within species, due in part to their location on the chromosome, and their ability to serve the cell not only through efflux of antibiotics, but also through efflux of heavy metals and other toxic compounds, secretion of virulence factors, and transport of compounds such as bile salts and fatty acids across the cell membrane.^51^ Given the wide utility of these ARGs, they are found in many environments and their detection is not likely concerning for human health, thus making them poor monitoring targets.

Despite the tendency of taxonomic diversity to decrease (Figure S2), the diversity of ARGs from influent to final effluent increased at all 12 WWTPs, confirming our hypothesis (5). This observation is consistent with the idea that biological processes, such as activated sludge, which is known to harbor a highly diverse range of ARGs,^52,53^ can seed additional ARGs to the final effluent. However, diversity indices represent only high-level trends, requiring closer examination of the behavior of individual ARGs and ARG classes. In particular, 22 ARGs were noted to be highly abundant in influent yet also experienced the greatest overall removal during treatment (Figure S3). Thus, in developing strategies for WWTP surveillance, such ARGs should be included to confirm expected treatment performance, while including ARGs that can be prone to persist or increase during treatment.

While the limited enrichment of only a handful of ARGs observed herein is inconsistent with the notion that WWTPs are a “hotspot” for the uncontrolled spread of ARGs, it nevertheless highlights the potential for a substantial number of clinically important ARGs to escape treatment. As new antibiotic resistant strains emerge, careful monitoring of ARGs in both WWTP influent and effluent can identify potentially clinically relevant ARG targets that may become enriched during biological treatment, or simply are released into the environment in significant numbers, which may prompt a closer examination of potential exposure pathways. Attention to pathogens carrying ARGs will be most informative of transmission risks.^54,55^ At the same time, it is important to recognize that metagenomic detection limits are relatively high^56^ and likely will not capture rare, but important, evolutionary events contributing to clinically important resistance.^21,57^ Further, metagenomic monitoring will detect DNA from inactivated organisms, which can still be useful if the goal is informing wastewater-based surveillance (WBS), but not as much so if the goal is assessment of transmission risks. For example, we suspect the detection of resistant *N. gonorrheae* would likely not reflect a viable pathogen.

The extensive information derived from metagenomic sequencing of WWTP samples yields insights into some of the key mechanisms driving observed resistome shifts through wastewater treatment. By far the dominant factor will be shifts in taxonomic hosts due to changing ecological conditions, making it difficult to discern contributions of HGT, selection, and coselection by antibiotics and other toxic compounds. While ARGs encoded chromosomally and on plasmids can both cause resistance in clinical infections, acquired or mobile ARGs (i.e., those documented to be transferred by HGT) are of particular concern given their ability to spread between species.^58^ Interestingly, while the mobile fraction of the resistome was reduced in relative abundance as a result of treatment across the 12 WWTPs, the abundance of MGE-associated ARGs increased. While wastewater treatment may drive increases in plasmids due to HGT or vertical transfer, this will not necessarily increase ARGs unless there is subsequent growth of the recipient.^59^ Several previous studies have demonstrated that microbial community composition strongly shapes environmental resistomes.^11,26,60^ This study further validated this result, finding that there was a moderate, but significant, correlation between taxonomic and resistome profiles from across the data set. Given that this study compiled data from 12 WWTPs from across the globe, the observed concordance between taxonomy and ARG profile is notable.

Antibiotics persisting throughout the wastewater treatment process have been identified as a potential selective force encouraging the preferential survival of ARB over nonresistant counterparts.^25,61^ Some of the measured antibiotic quantities were in excess of predicted no effect concentrations (PNEC), suggesting that there is potential for selection within the wastewater environment.^62^ For example, 92% of influent samples and 58% of effluent samples were in excess of 64 ng/L of the quinolone antibiotic ciprofloxacin, 33% of influent samples and 17% of effluent samples were in excess of 250 ng/L of the macrolide antibiotic clarithromycin, and 17% of influent samples and 8% of effluent samples were in excess of 500 ng/L of the quinolone antibiotic norfloxacin. The percentage of samples in excess of the PNEC for erythromycin (8%) and azithromycin (17%) remained the same from influent to effluent.

This study demonstrates that it is important to examine multiple targets of antibiotic resistance in monitoring campaigns and that it may be misleading to summarize resistome data via a single summary statistic, such as overall removal of total ARGs. While metagenomic sequencing for wastewater resistome characterization is the most comprehensive approach for ARG monitoring, metagenomic sequencing could also be valuable for informing selection of potential ARG monitoring targets.^63^ It is important to optimize sampling and monitoring strategies based on specific objectives and to ensure that monitoring targets are selected accordingly.^64^ Potential end goals include:^65^ (1) characterizing the prevalence of ARB in a given human population via WWTP influent or sewage collection system monitoring (i.e., WBS),^13,14,66^ (2) monitoring for the risks of evolution of new pathogenic strains of resistant organisms, also involving selection and HGT during biological treatment processes,^67,68^ (3) assessing the effectiveness of treatment processes for ARG removal via sampling of WWTP effluent, often alongside influent,^27,69^ and (4) assessing potential human health risks associated with exposures, particularly to resistant pathogens, downstream of WWTP effluent discharge via sampling receiving water bodies.^70,71^ In WBS and effluent monitoring for human health risk applications, it is important to select clinically relevant ARGs that are associated with pathogens linked to human infection. Linking ARGs to specific hosts is thus necessary. To identify selection pressures that could drive resistance evolution within WWTPs, simple analyses of relative abundances of ARGs is insufficient since increases in ARGs can be caused by taxonomic shifts unrelated to the antibiotic selection pressure.

Monitoring for the emergence of new strains presents numerous challenges. The use of scaffolds assembled from short reads helped identify potential ARG host bacteria. However, the taxonomic origin of ARGs may be impacted by the ability of conjugative transposons to facilitate movement of chromosomal genes to plasmids. When monitoring effluent to assess treatment process effectiveness, ARGs that are prevalent in influent as well as effluent, such as *sul*1, are strong candidates, though targets that are also prevalent in natural environments and have high background concentrations should be excluded. For all of these goals, metagenomic sequencing is valuable for assessing broad changes in the resistome that may be overlooked when narrowly selecting monitoring targets. When or where a metagenomics approach is not yet feasible or practical, the results of this and other metagenomic surveys can be useful for identifying potential monitoring targets.

## Supporting Information Available

Characteristics of sampled WWTPs; primers and cycling conditions used for qPCR; abundances of the sul1 resistance gene via qPCR versus metagenomics; Shannon diversity in the influent versus final effluent at each WWTP calculated for ARGs and taxonomic classification at the genus level; subset of ARGs that fell within both the 15th percentile of highest influent concentrations and the 15th percentile of reductions in relative abundance during treatment; discriminatory ARGs in influent vs final effluent; key assembled scaffolds of interest; abundance of MGEs; concentrations of antibiotics measured in collected samples; relative abundance of ARGs and corresponding classes of antibiotics in influent versus final effluent; microbial taxonomy in influent and effluent; symmetric Procrustes analysis between microbial community and resistome; percentage mobility (% M) among samples (PDF)
Relative abundance of all ARGs across all samples as determined by metagenomic sequencing; all annotations of ARGs, MGEs, and metal resistance genes on assembled scaffolds; frequency of detection of ARGs on assembled scaffolds; percentage mobility (% M) among ARGs; count of co-occurring scaffold-associated ARGs (XLSX)
