# Supplementary material for: Metagenomic Profiling of Internationally Sourced Sewage Influents and Effluents Yields Insight into Selecting Targets for Antibiotic Resistance Monitoring
Source: Environ Sci Technol. 2024 Sep 4;58(37):16547–59. doi: 10.1021/acs.est.4c03726 (PMC11411718; doi:10.1021/acs.est.4c03726)
Supplement: Supplementary file 1 — es4c03726_si_001.pdf [file es4c03726_si_001.pdf]

Supplementary Information

Title: Metagenomic Profiling of Internationally Sourced Sewage Influents and Effluents Yields Insight into Selecting Targets for Antibiotic Resistance Monitoring

Emily Garner[a,b], Ayella Maile-Moskowitz[b], Luisa F. Angeles[c], Carl-Frederick Flach[d], Diana S. Aga[c], Indumathi Nambi[e], D. G. Joakim Larsson[d], Helmut Burgmann[f], Tong Zhang[g], Peter J. Vikesland[b], Amy Pruden[b]

[a] Wadsworth Department of Civil and Environmental Engineering, West Virginia University, Morgantown, West Virginia, 26505, USA

[b] Department of Civil and Environmental Engineering, Virginia Tech, Blacksburg, Virginia, 24061, USA

[c] Department of Chemistry, University at Buffalo, New York, 14260, USA

[d] Institute of Biomedicine, Department of Infectious Diseases, University of Gothenburg, Sweden and Centre for Antibiotic Resistance Research in Gothenburg (CARe), Västra Götaland, SE-405 30 Gothenburg, Sweden

[e] Department of Civil Engineering, Indian Institute of Technology, Madras, Chennai, 600036, India

[f] Eawag: Swiss Federal Institute of Aquatic Science and Technology, Kastanienbaum, CH-6047, Switzerland

[g] Department of Civil Engineering, The University of Hong Kong, Pokfulam, Hong Kong

11 pages: 3 tables, 10 figures, 5 captions for supplementary datasheets

Table S1: Characteristics of sampled wastewater treatment plants

**Treatment Plant ID**	**Sampling Date**	**Sewage Composition**	**Treatment Configuration**
CHE-1	May 17, 2016	90% municipal, 10% industrial	Conventional activated sludge
CHE-2	May 18, 2016	50% municipal, 50% industrial	Phosphate/Fe precipitation after primary settling, Conventional activated sludge, ozone disinfection
HKG-1	July 14, 2016	Municipal Sewage	Mechanical bar screens and degritting, conventional activated sludge and sedimentation, ultraviolet disinfection
HKG-2	July 14, 2016	Municipal sewage + treated seawater	Conventional activated sludge, ultraviolet disinfection
IND-1	March 10, 2016	Municipal Sewage	Activated sludge with diffused aerators, chlorination without dechlorination
IND-2	March 10, 2016	Municipal Sewage	Activated sludge with mechanical mixers, chlorination
PHL-1	December 2, 2016	Municipal sewage + industrial input	Attached-growth bioreactor with aeration basin as polishing step, chlorination
PHL-2	November 29, 2016	Municipal Sewage	Conventional activated sludge
SWE-1	June 8, 2016	Municipal sewage + hospital and industrial input	70% conventional activated sludge, 30% trickling filters
SWE-2	June 9, 2016	Municipal sewage + hospital and industrial input	Conventional activated sludge
USA-1	November 1, 2016	95% municipal	2-stage activated sludge, ultraviolet disinfection
USA-2	January 19, 2017	80% municipal, 20% industrial	Step-feed activated sludge (nitrification), down flow denitrifying filters, chlorination

Table S2: Primers and cycling conditions used for quantitative polymerase chain reaction (qPCR)

Gene target	Primer Sequences	Cycling Conditions	Reference
Universal 16S rRNA genes	1369F: 5'-CGGTGAATACGTTCYCGG-3'<br>1492R: 5'-GGWTACCTTGTTACGACTT-3'	50°C for 2 min, 95°C for 2 min, 40 cycles of (95°C for 5 s, 55°C for 30 s, 72°C for 30 s), melt curve from 60 to 95°C	[1]
*sul*1	RW: 5'-CGCACCGGAAACATCGCTGCAC-3'<br>RV: 5'-TGAAGTTCCGCCGCAAGGCTCG -3'	95°C for 2 min, 50 cycles of (95°C for 15 s, 65°C for 30 s, 72°C for 30 s), melt curve from 60 to 95°C	[2]

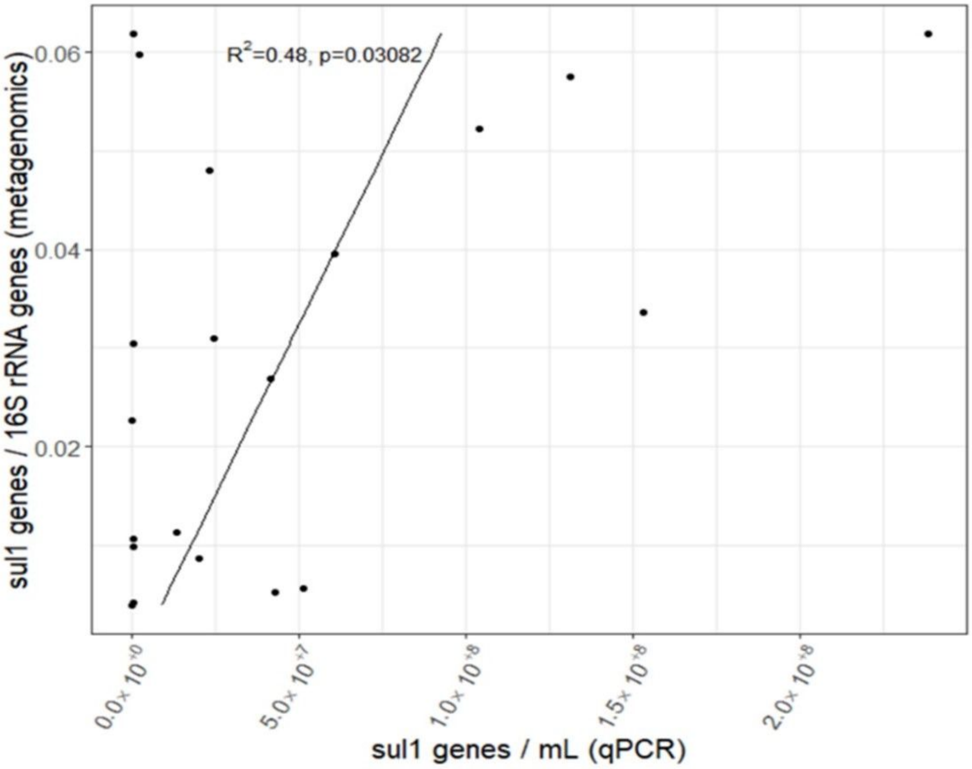

Figure S1: Abundances of the *sul*1 resistance gene are correlated when determined via qPCR versus metagenomics.

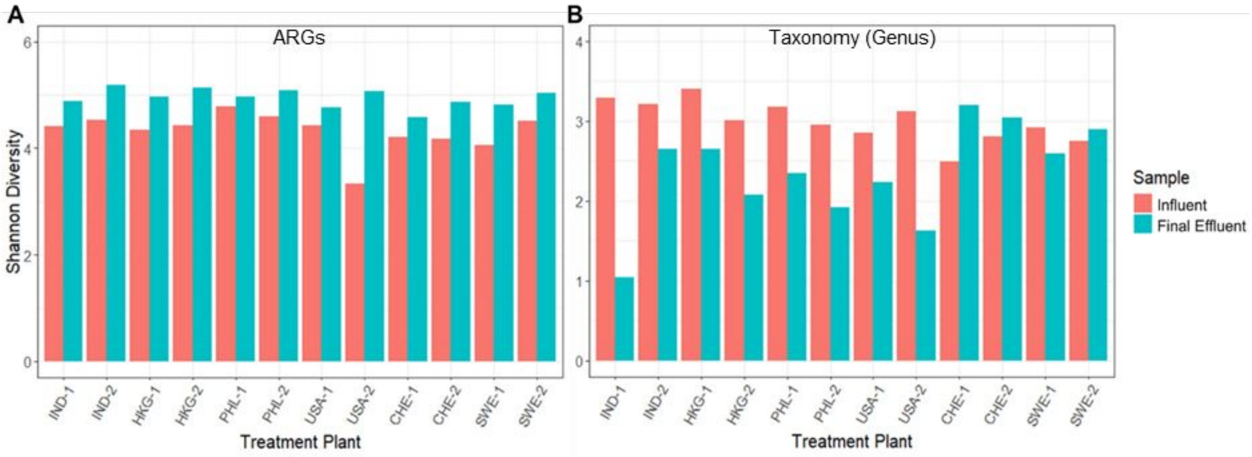

Figure S2: Shannon diversity in the influent versus final effluent at each WWTP calculated for (A) ARGs and (B) taxonomic classification at the genus level.

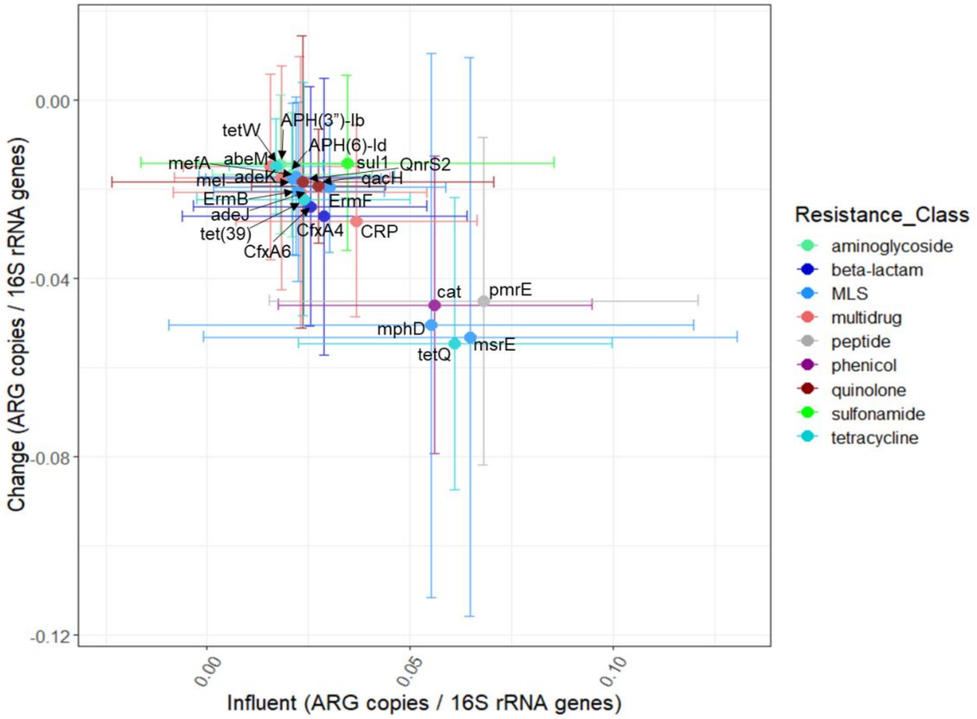

Figure S3: A portion of ARGs examined in this study that were highly abundant in influent were also among those that experienced the highest overall removal during treatment. To identify the key contributors to this trend, ARGs that fell within both the 15th percentile of highest influent concentrations and the 15th percentile of reductions in relative abundance during treatment were identified.

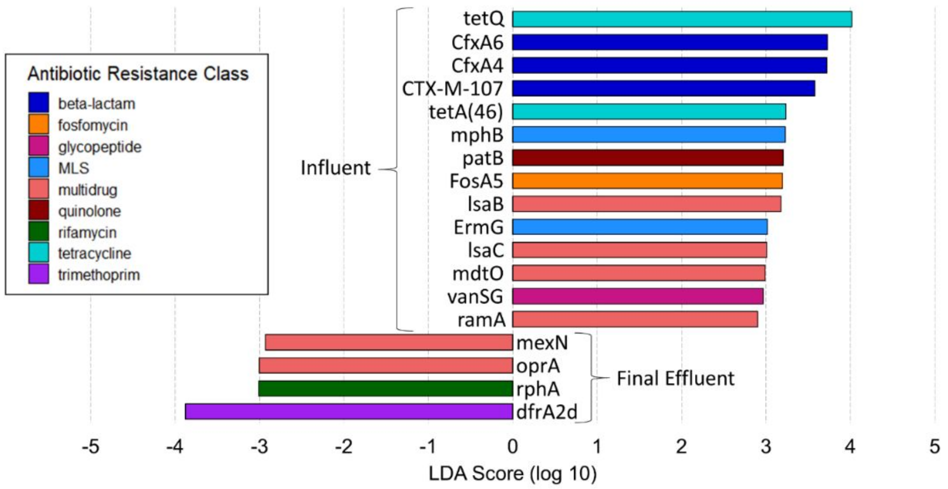

<u>Figure S4</u>: Discriminatory ARGs in influent vs final effluent, determined by Linear discriminant analysis Effect Size (LEfSe).

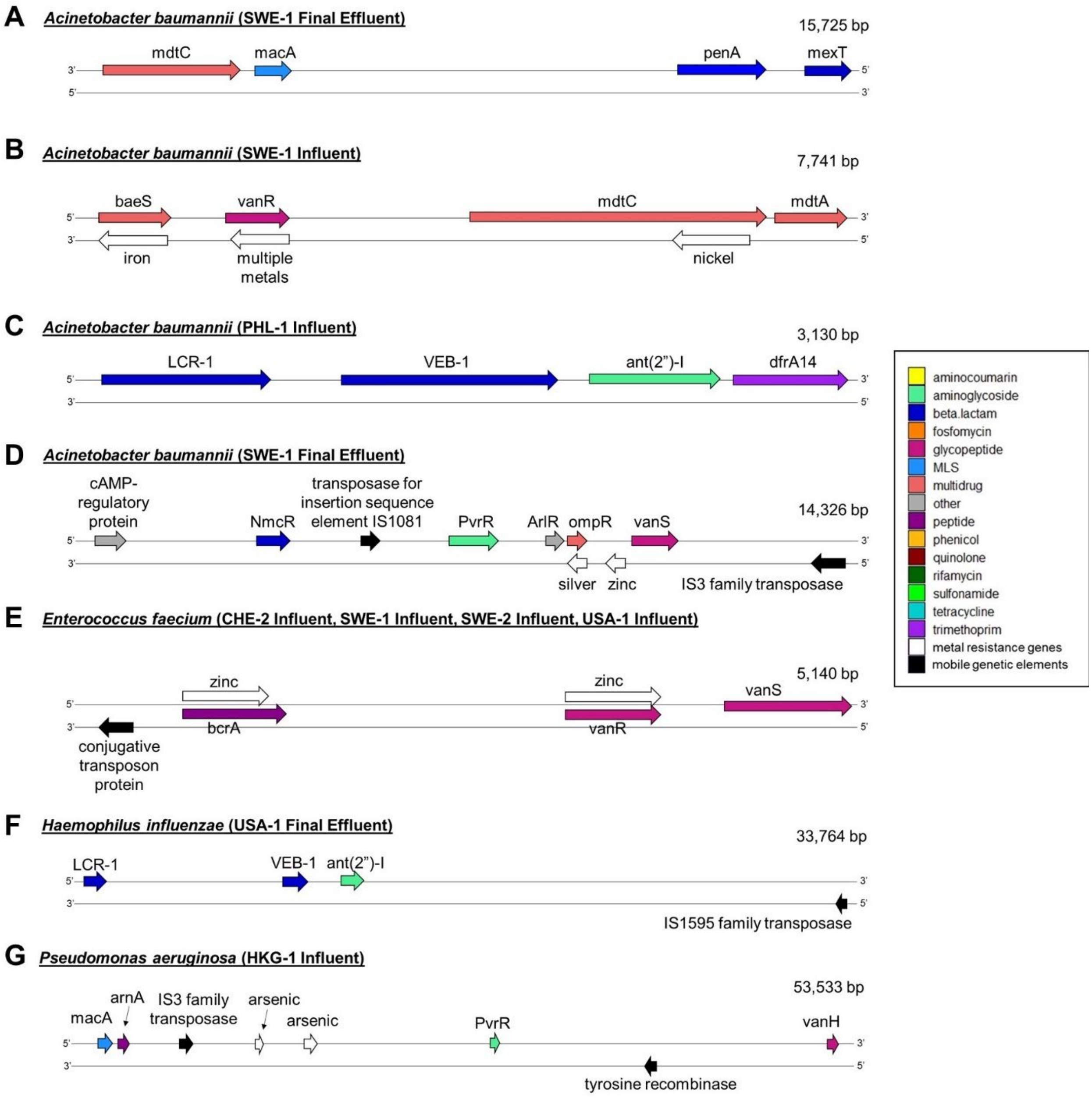

Figure S5: Key assembled scaffolds of interest. Scaffolds were selected based on origination from a pathogen, presence of three or more ARGs, presence of mobile genetic elements and metal resistance genes, and repeated detection of conserved regions across multiple samples.

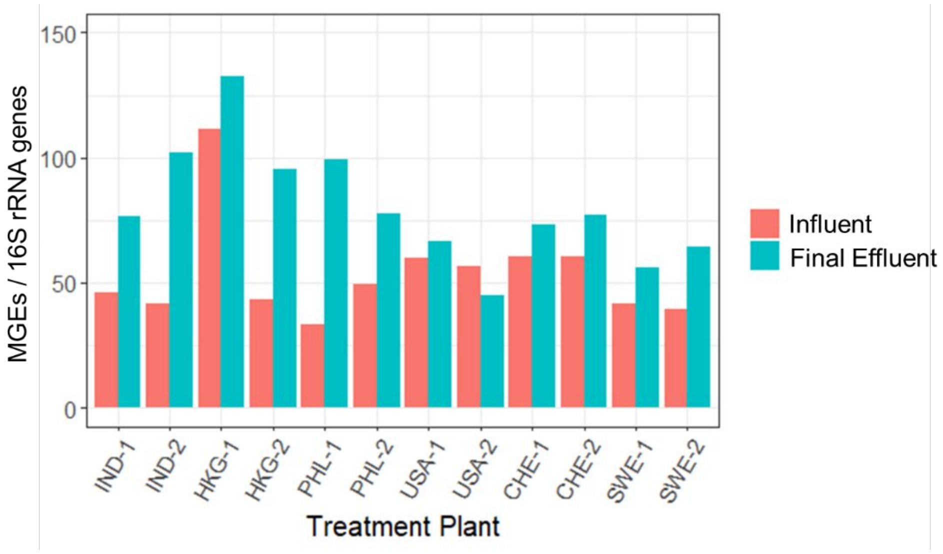

<u>Figure S6</u>: Abundance of MGEs normalized to 16S rRNA genes.

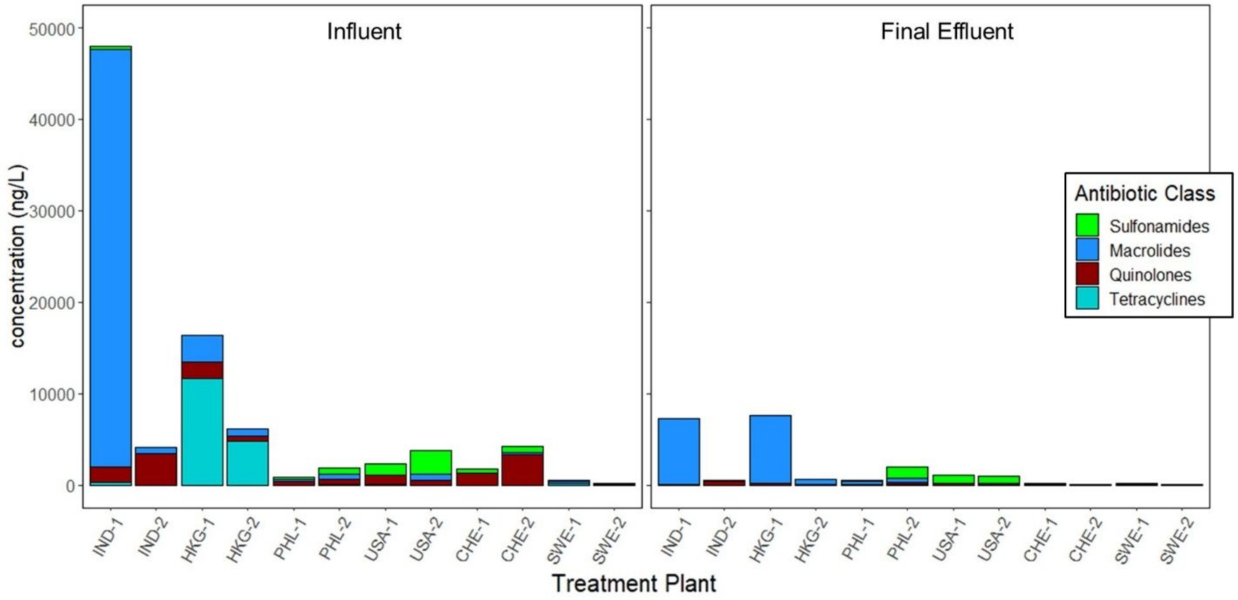

<u>Figure S7</u>: Concentrations of antibiotics measured in collected samples. Note: Samples from Indian WWTPs were collected in 2017, one year after samples were collected for metagenomic analysis.

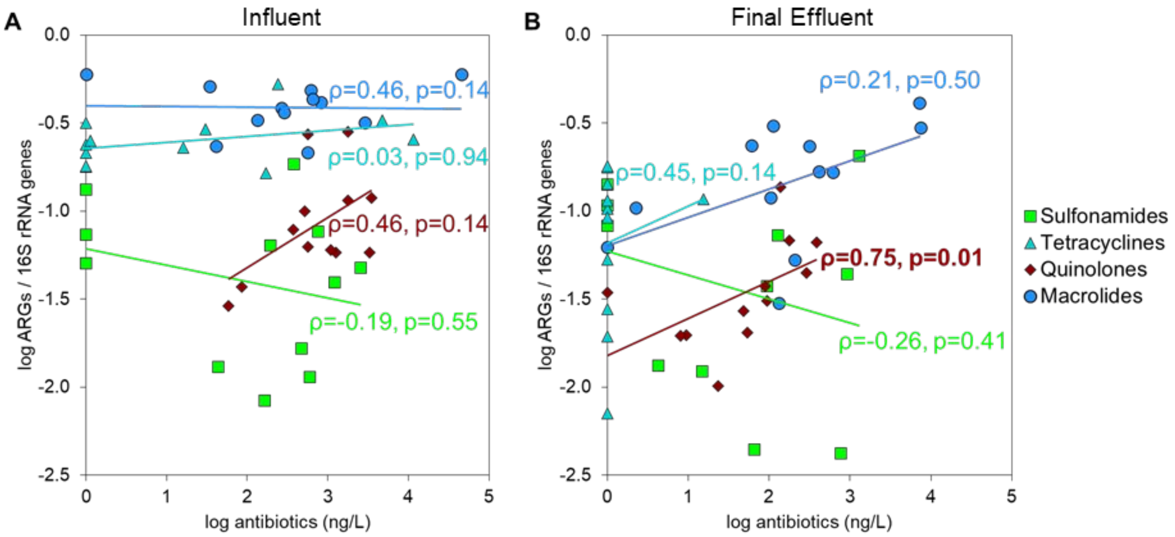

<u>Figure S8</u>: Relative abundance of ARGs and corresponding classes of antibiotics in (A) influent versus (B) final effluent. Significant correlations as determined by Spearman's rank sum correlation test are indicated in bold. The only statistically significant correlation occurred between quinolone antibiotics and ARGs in final effluent. Though not statistically significant, quinolone antibiotics and ARGs were also correlated in the influent, suggesting that this may be an instance of upstream selection in intestinal bacteria and subsequent co-persistence of quinolone antibiotics and ARGs present in the influent, rather than selection. This hypothesis is further supported by the fact that the absolute abundance of quinolone ARGs (per unit volume) decreased at 11 of 12 WWTPs.

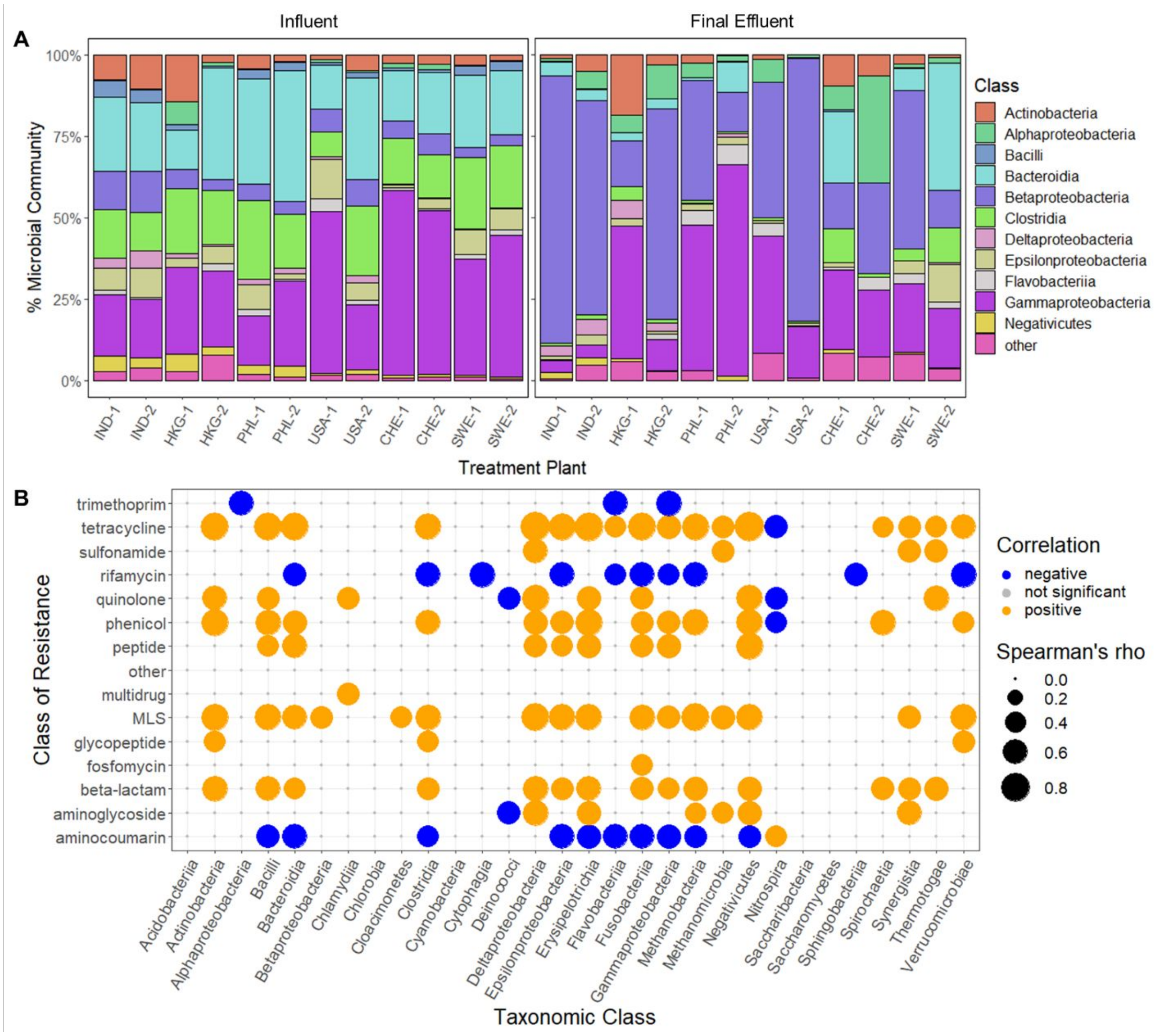

Figure S9: A) Classes of bacteria as a percentage of the overall microbial community across the WWTPs based on annotation of reads. B) Statistically significant correlations (according to Spearman's rank sum correlation test) between classes of bacteria and classes of resistance among annotated reads.

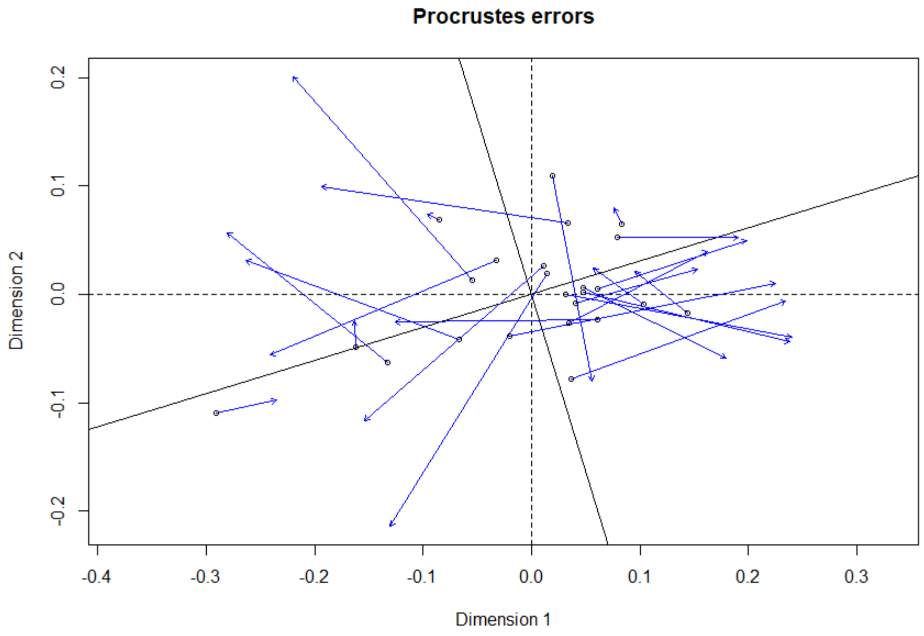

Figure S10: Symmetric Procrustes analysis between microbial community (circles) and resistome (ends of biplots). Procrustes Sum of Squares (m_{12}^2) = 0.7326, Correlation in a symmetric Procrustes rotation = 0.5171, Significance = 0.004.

Table S3: Relative abundance (normalized to 16S rRNA gene) of all ARGs across all samples, as determined by metagenomic sequencing (provided as supplementary dataset)

Table S4: All annotations of ARGs, MGEs, and metal resistance genes on assembled scaffolds (provided as supplementary dataset)

Table S5: Frequency of detection of ARGs on assembled scaffolds (provided as supplementary dataset)

Table S6: Percentage mobility (%M) among samples

Sample	Total scaffolds with ARGs	Total scaffolds with ARGs and MGEs	%M
CHE-1 FE	879	107	12.17292
CHE-2 FE	2720	342	12.57353
HKG-1 FE	3078	367	11.92333
HKG-2 FE	1001	131	13.08691
IND-1 FE	3959	502	12.67997
IND-2 FE	3004	361	12.01731
PHL-1 FE	1838	243	13.22089
PHL-2 FE	3183	386	12.12692
SWE-1 FE	2320	314	13.53448
SWE-2 FE	605	60	9.917355
USA-1 FE	770	101	13.11688
USA-2 FE	1408	170	12.07386
CHE-1 IN	2784	364	13.07471
CHE-2 IN	4776	572	11.97655
HKG-1 IN	2900	379	13.06897
HKG-2 IN	4518	574	12.70474
IND-1 IN	2977	391	13.13403
IND-2 IN	3866	508	13.1402
PHL-1 IN	3864	483	12.5000
PHL-2 IN	4118	479	11.63186
SWE-1 IN	3599	419	11.64212
SWE-2 IN	4268	494	11.57451
USA-1 IN	1228	167	13.59935
USA-2 IN	2973	340	11.43626

Table S7: Percentage mobility (%M) among ARGs (provided as supplementary dataset)

Table S8: Count of co-occurring scaffold-associated ARGs (provided as supplementary dataset)

References

(1) Suzuki, M. T.; Taylor, L. T.; DeLong, E. F. Quantitative Analysis of Small-Subunit rRNA Genes in Mixed Microbial Populations via 5′-Nuclease Assays. *Appl. Environ. Microbiol.* **2000**, *66* (11), 4605–4614. https://doi.org/10.1128/AEM.66.11.4605-4614.2000.

(2) Pei, R.; Kim, S.-C.; Carlson, K. H.; Pruden, A. Effect of River Landscape on the Sediment Concentrations of Antibiotics and Corresponding Antibiotic Resistance Genes (ARG). *Water Research* **2006**, *40* (12), 2427–2435. https://doi.org/10.1016/j.watres.2006.04.017.
